# Intracardiac echocardiography improves lesion quality and ablation efficiency of pulmonary vein isolation in atrial fibrillation patients: a propensity score-matched analysis

**DOI:** 10.3389/fcvm.2025.1612181

**Published:** 2025-07-01

**Authors:** Ye Deng, Li Deng, Qingqing Gu, Qianwen Chen, Yang Zhang, Jun Wei, Xu Liu, Yuan Ji, Ling Sun, Qingjie Wang

**Affiliations:** ^1^ Department of Cardiology, The Third Affiliated Hospital of Nanjing Medical University, Changzhou, Jiangsu, China; ^2^ Department of Cardiology, The Affiliated Wuxi People’s Hospital of Nanjing Medical University, Wuxi, Jiangsu, China; ^3^ Department of Cardiovascular Surgery, The Affiliated Hospital of Xuzhou Medical University, Xuzhou, China; ^4^ Department of Research and Development, Johnson & Johnson (Shanghai) Medical Equipment Co, Ltd, Shanghai, China

**Keywords:** atrial fibrillation, catheter ablation, pulmonary vein isolation, intracardiac echocardiography, recurrance

## Abstract

**Background:**

Pulmonary vein isolation (PVI) is a cornerstone of catheter ablation for atrial fibrillation (AF). Intracardiac echocardiography (ICE) offers real-time imaging that may enhance procedural outcomes compared to traditional x-ray guidance. This study evaluates the impact of ICE on PVI lesion quality and efficiency using a novel Ablation Index Functional Validation (AIFV) system.

**Methods:**

This single-center, retrospective, matched cohort study included AF patients undergoing catheter ablation between June 2022 and June 2023 at The Third Affiliated Hospital of Nanjing Medical University. Patients were grouped based on ICE use (ICE vs. No-ICE), with intraoperative data recorded via the VisiTag system and analyzed by AIFV. Propensity score matching (1:1) was applied to compare procedural efficiency and lesion quality (primary endpoints) and AF recurrence (secondary endpoint) between groups.

**Results:**

Of 126 patients enrolled (61 ICE, 65 No-ICE), 46 matched pairs were analyzed. PVI was achieved in all cases without severe complications. The ICE group demonstrated significantly shorter total PVI time [2,819 s (2,565 s, 2,953 s) vs. 3,153 s (2,696 s, 3,831 s), *p* = 0.006], higher radiofrequency (RF) time ratio (59.1% ± 13.9% vs. 48.2% ± 11.6%, *p* < 0.001), and higher effective ablation-index (AI) ratio (96.1% ± 4.5% vs. 91.2% ± 3.9%, *p* < 0.001) compared to the No-ICE group. Left and right PVI times were also reduced (*p* = 0.034 and *p* = 0.029, respectively). At 12-month follow-up, AF recurrence rates were significantly lower in the ICE group (7.7% vs. 30.8%, *p* = 0.038) in persistent AF patients.

**Conclusion:**

ICE enhances the quality of lesions and the ablation efficiency of PVI in AF patients, as shown by the AIFV system.

## Graphical Abstract

**Figure:**
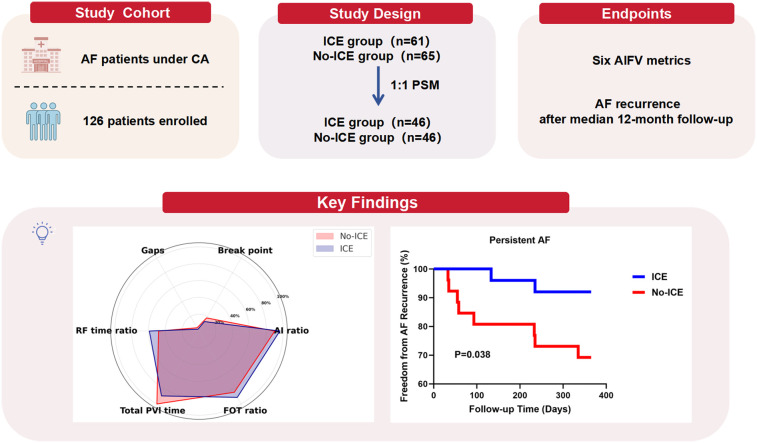
Graphical abstract of our finding.

## Introduction

Atrial fibrillation (AF) is the most prevalent arrhythmia encountered in clinical practice, significantly increasing the risk of stroke, heart failure, and other cardiovascular complications (1). It is projected that by 2050, approximately 9 million individuals aged 60 and above in China will be affected by AF. Current guidelines recommend catheter ablation as the first-line approach for rhythm restoration in patients with symptomatic AF and pulmonary vein isolation (PVI) serves as the fundamental approach in the ablation (1, 2). Enhancing lesion quality and securing consistent, transmural, and lasting PVI lesions are critical for maximizing single-procedure success rates (3). Significant advancements have been made in ensuring stable catheter contact and transmural lesions, including techniques such as high-frequency intubation, steerable long sheaths, and force-detection catheters (1). Intracardiac echocardiography (ICE), a novel technology introduced in recent years, enables real-time, two-dimensional visualization of the heart during ablation procedures (4–7). When integrated with three-dimensional (3D) mapping systems, ICE enables the creation of three-dimensional models, providing more accurate and intuitive localization, reducing unnecessary catheter movement during the procedure and potentially enhancing the efficacy of AF ablation (8–10). Previous studies have established ICE's utility in procedural guidance, such as left atrial appendage closure and phrenic nerve visualization (8, 9), but its specific impact on PVI lesion quality and efficiency compared to traditional x-ray guidance is less explored. In this study, we employed a novel Ablation Index Functional Validation (AIFV) system, consisting of six key criteria, to thoroughly assess PVI quality and ablation efficiency using ICE in comparison to x-ray guidance in patients with AF (11).

## Methods

### Study design and population

This was a single-center, retrospective, matched cohort study. Patients experiencing symptomatic AF who had their initial ablation procedure using ICE at The Third Affiliated Hospital of Nanjing Medical University between June 2022 and June 2023 were included in the study. For comparison, a control group was formed of patients who underwent ablation during the same timeframe under x-ray guidance, performed by the same team of physicians. Consistent with current guidelines, AF was classified as paroxysmal (self-terminating within 48 h) or persistent (lasting >7 days, including cases requiring cardioversion thereafter). The inclusion criteria were as following: (1) adults aged 18–80 years; (2) patients examined with 12-lead electrocardiogram (ECG) or Holter monitoring for AF; (3) patients with persistent and paroxysmal AF; (4) patients having not taken class I or III antiarrhythmic drugs. The exclusion criteria were as follows: (1) patients with a previous ablation history, (2) patients with structural cardiac disorders, (3) patients with end stage renal disease (ESRD) and (4) unwillingness to follow-up.

### Catheter ablation of atrial fibrillation with ICE

All patients received novel oral anticoagulants for a minimum of three weeks prior to ablation (12). Transesophageal echocardiography (TEE) was routinely performed to exclude left atrial thrombus before the procedure (13–16). Anti-arrhythmic medications were stopped at least five half-lives prior to ablation (3).

**Figure 1:**
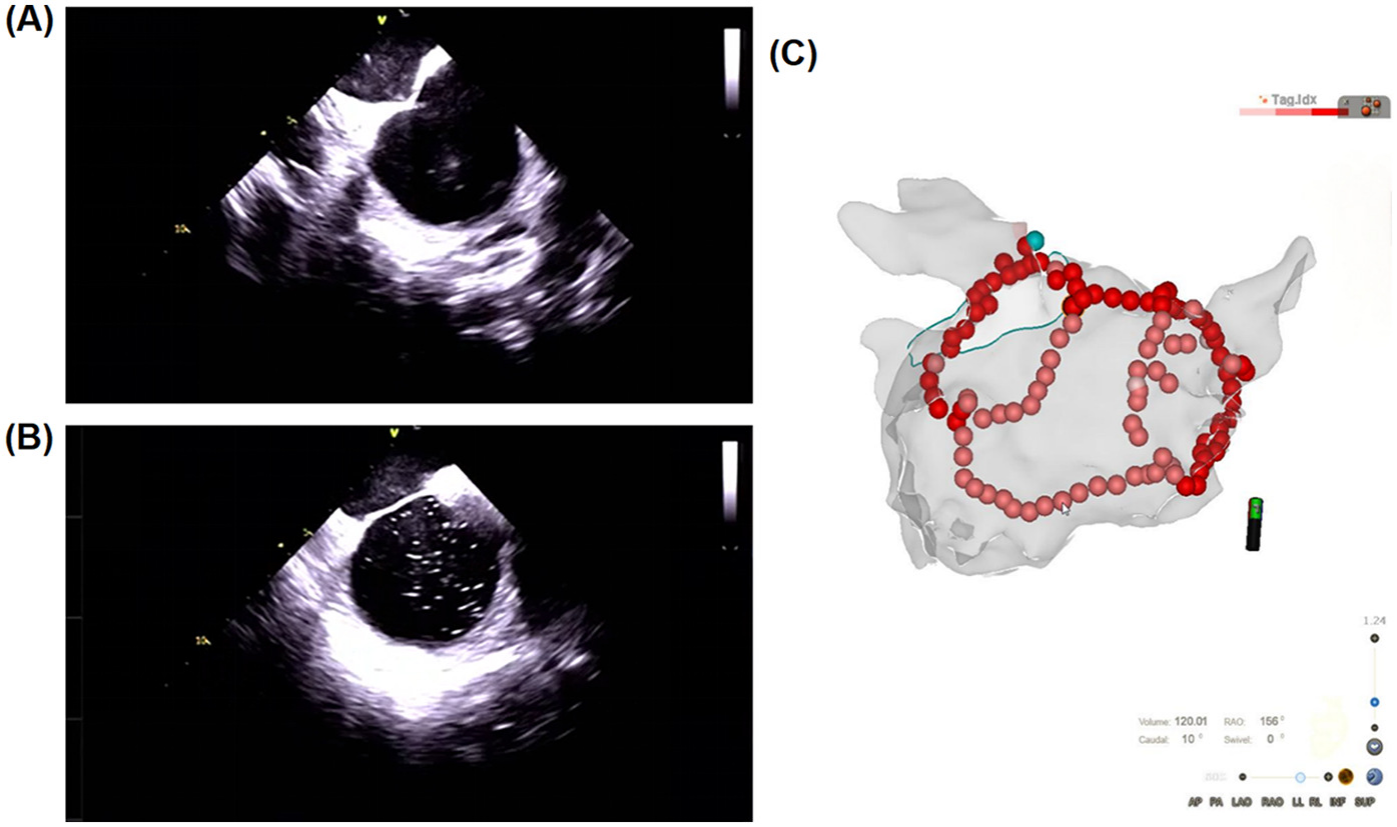
Typical images of catheter ablation of atrial fibrillation with ICE. **(A)** “Tent sign”: Atrial septum being tented by the tip of the transseptal puncture needle. **(B)** “Bubble sign”: As the puncture needle passes through the atrial septum, “tent sign” disappears and the infusion of saline reveals the “bubble sign” in the left atrium. **(C)** Ablation points recorded by Visitag system combined with atrial three-dimensional anatomical shell guided by ICE and CARTO3 system.

During the procedure, a 10-F, 65-cm ICE sheath was advanced into the right atrium to identify key anatomical landmarks, including the tricuspid annulus, coronary sinus ostium, superior vena cava, and atrial septum. An additional 8.5-F, 65-cm sheath was introduced into the left atrium through a standard transseptal puncture. Successful puncture was confirmed by the “tent sign” and “bubble sign,” as shown in Figure 1. After the transseptal puncture, heparin was administered intravenously to ensure the activated clotting time remained within the target range of 300–350 s (8, 17). A 3D anatomical model of the atrium was created with a mapping catheter using ICE and the CARTO3 system for guidance. Low-voltage areas were identified using a PentaRay multipolar mapping catheter (Biosense Webster Inc.) integrated with the CARTO3 system, enabling high-resolution mapping of the left atrium to detect regions of atrial fibrosis or scar. An Agilis NxT steerable sheath (Abbott) was used in all procedures for both the ICE and No-ICE groups to enhance catheter stability and maneuverability, ensuring consistency in lesion formation across operators. The complete procedure was conducted by a trio of seasoned physicians, each having performed over 200 AF ablation procedures and more than 30 cases with the SmartTouch catheter.

A standardized approach was adopted for AF ablation. PVI was achieved using an ablation-index (AI)-guided technique with a SmartTouch catheter, delivering power settings between 30 and 45 watts and maintaining contact forces ranging from 5 to 30 grams. A point-by-point radiofrequency (RF) application was employed to form a continuous circular lesion, with each tag measuring 4 mm in diameter. Target AI values were established at 500 for the anterior segments, 400–450 for the roof, and 350–400 for the inferior and posterior segments (11). In cases where AF continued post-PVI, electrical cardioversion was performed to reestablish sinus rhythm. Furthermore, if low-voltage regions were identified during left atrial voltage mapping, additional substrate ablation was carried out. All procedures were performed by the same team of experienced physicians (>200 AF ablations each).

### Evaluation of procedure parameters by AIFV system

**Figure 2:**
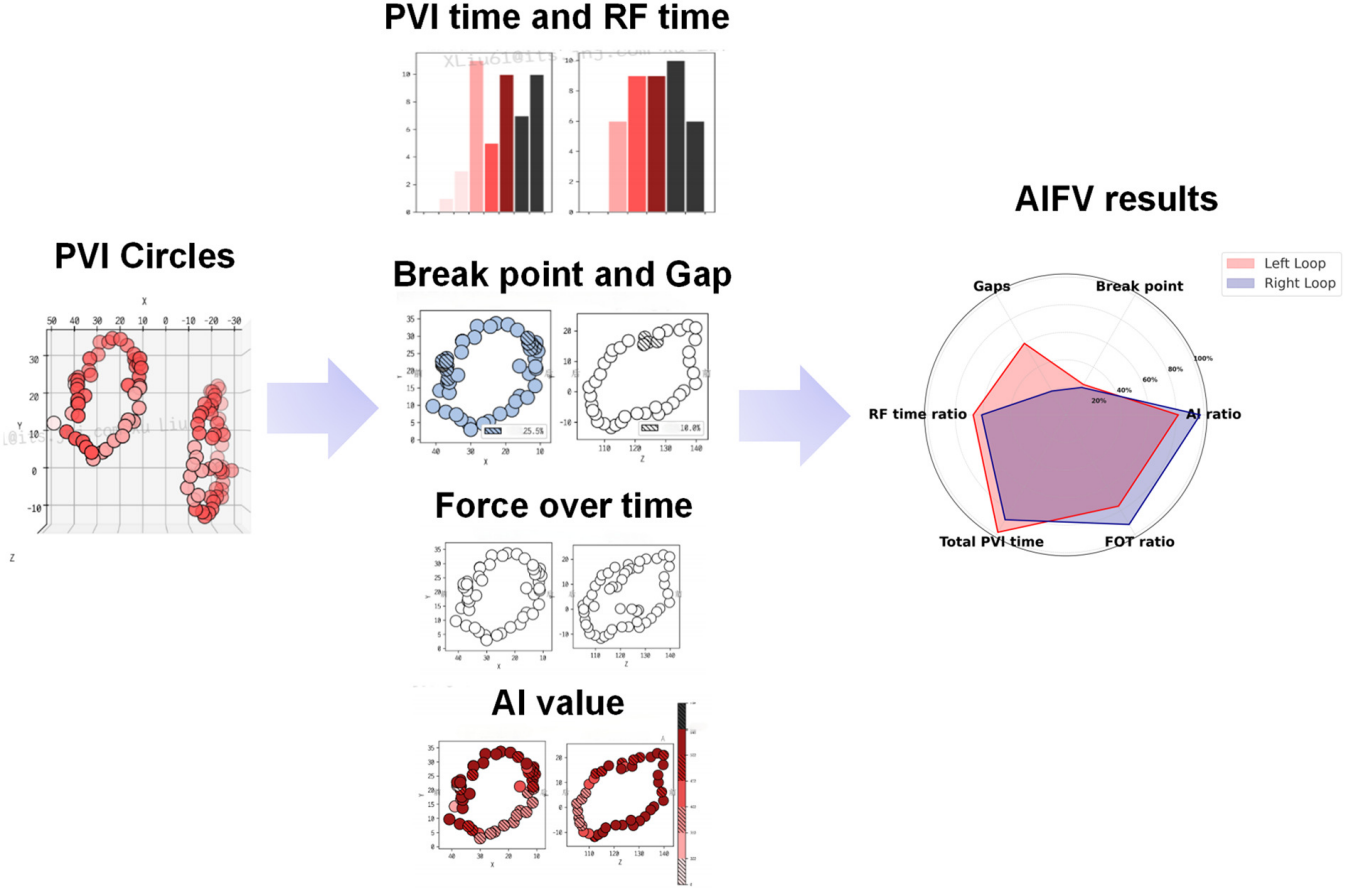
An example of AIFV results of AF patients after CA. The AIFV system will independently evaluate each ablation point in pulmonary vein (PV) circles, including inter-lesion distance, percentage of lesions with effective force-over-time (FOT ratio), percentage of lesions with effective AI (AI ratio), radiofrequency time, etc., and finally present the results in the form of a hexagonal radar chart.

Ablation points on the 3D mapping system were documented using the VisiTag system (VisiTagR, Biosense Webster Inc.), which records catheter position, stability (≤2.5 mm motion), duration (>3 s), and contact force (≥5 g for ≥25% of the time) during ablation, as shown in Figure 1 (11). These data were integrated with a 3D anatomical model created using the CARTO3 system. The Ablation Index Functional Validation (AIFV) system (Biosense Webster Inc., Irvine, CA) is a proprietary software tool designed to quantitatively assess the quality and efficiency of PVI by processing VisiTag data. The AIFV system evaluates each ablation point within the pulmonary vein circles based on six key metrics: (a) Break Point: the percentage of inter-lesion distances ≤3 mm, indicating lesion continuity; (b) Gap: the number of inter-lesion distances ≥6 mm, reflecting potential gaps in the ablation line; (c) Effective Force-Over-Time (FOT) Ratio: the percentage of lesions with a contact force >5 g for >40% of the ablation time; (d) Effective Ablation Index (AI) Ratio: the percentage of lesions with AI values between 300 and 600, indicating effective energy delivery; (e) Total PVI Time: the duration from the first to the last radiofrequency (RF) application during PVI; and (f) RF Time Ratio: the proportion of total PVI time spent on RF applications. The AIFV system generates a statistical summary of these metrics and presents them in a hexagonal radar chart for enhanced visualization, as shown in Figure 2. While the specific algorithms used by AIFV are proprietary, the metrics are grounded in established ablation principles. Intraoperative data were prospectively collected during ablation, and AIFV analysis was performed retrospectively by an independent team blinded to group allocation to ensure objectivity.

### Follow-up

All patients received antiarrhythmic drugs (AADs) for a duration of three months, granted there were no contraindications, and were subsequently monitored in an outpatient clinic following the ablation procedure (18). Clinical assessments and 24-hour Holter recordings took place at intervals of 3, 6, 9, and 12 months post-procedure. Additionally, a 12-lead ECG was conducted whenever patients experienced arrhythmic symptoms.

### Endpoints

In this study, the primary endpoint was total PVI time, defined as the duration from the first to the last radiofrequency (RF) application during PVI, reflecting procedural efficiency. Additional measures of lesion quality, assessed via AIFV metrics (e.g., RF time ratio, effective AI ratio, effective FOT ratio, break point ratio, and gap number), were evaluated as co-primary endpoints. The secondary endpoint was AF recurrence, defined as episodes lasting >30 s after a 3-month blanking period, assessed over a 12-month follow-up via ECG and Holter monitoring (19).

### Sample size calculation

This study is a retrospective analysis, and the sample size was determined by the available clinical data (61 patients in the ICE group and 65 in the No-ICE group). To evaluate the statistical power of our cohort to detect differences in the primary endpoint (total PVI time), we performed a *post hoc* power calculation. Based on prior literature, we assumed a mean total PVI time of 3,200 s in the No-ICE group and estimated that ICE could reduce this to 2,800 s (a 400-second difference). With *α* = 0.05 and a power of 80% (*β* = 0.2), a two-sample t-test indicated that 45 patients per group were required. After propensity score matching, 46 matched pairs were analyzed, providing adequate power to detect the hypothesized difference in PVI time.

### Statistical analysis

SPSS software (Version 27, IBM) was used. Continuous variables were reported as the mean ± SD or Median (25th percentile, 75th percentile), as appropriate. Categorical variables were expressed as numbers and percentages. Propensity score matching analysis was employed to pair each patient in the ICE group with a corresponding patient in the No-ICE group at a ratio of 1:1, using a caliper of 0.2 without replacements. The Student t-test or Mann–Whitney U test was performed to compare continuous variables in two groups, according to the results of normality test. The *χ*^2^ test or Fisher's exact test was used to compare categorical variables. After propensity score matching, a multivariate regression analysis was performed to assess the association between ICE use and key procedural outcomes (total PVI time, RF time ratio, effective AI ratio, and FOT ratio), adjusting for potential residual confounders, including age, gender, AF type, and LAD. All tests were two-sided, and a *P* value < 0.05 was considered statistically significant.

## Results

### Baseline characteristics

Between June 2022 and June 2023, a total of 61 patients who had ablation procedures under ICE were included in the ICE group. Additionally, 65 patients who underwent AF ablation under x-ray guidance by the same team of physicians during the same period were included in the No-ICE group. The baseline characteristics, laboratory test results and echocardiography parameters of the enrolled patients are summarized in Tables 1, 2. Patients in the ICE group had a greater incidence of persistent AF, symptomatic heart failure, hypertension, and alcohol use. Moreover, they demonstrated lower left ventricular ejection fraction (LVEF) and fractional shortening (FS), along with higher B-type natriuretic peptide (BNP) levels. All in all, cardiac function in the ICE group patients was markedly poorer. Propensity score matching was carried out using the following baseline variables: age, gender, types of AF, and several echocardiography parameters. Post-propensity score matching, a total of 92 patients (46 from the ICE group and 46 from the No-ICE group) were eligible for further analysis of procedural parameters. There was no significant disparity between these two groups in terms of baseline characteristics, laboratory test outcomes, and echocardiography parameters, as summarized in Tables 1, 2.

**Table 1:** Baseline characteristics of enrolled patients before and after propensity score matching.

Variables	Before matching			After matching		
	ICE (*n* = 61)	No-ICE (*n* = 65)	*P*	ICE (*n* = 46)	No-ICE (*n* = 46)	*P*
AGE (year)	69.0 (61.0, 73.0)	68.0 (62.0, 75.5)	0.508	69.43 ± 8.52	67.26 ± 6.59	0.339
Male, *n* (%)	43 (70.5)	36 (55.4)	0.08	34 (73.9)	24 (52.2)	0.127
BMI (kg/m^2^)	24.84 (23.29, 27.55)	25.86 (23.85, 27.34)	0.38	25.21 ± 3.91	25.41 ± 3.63	0.864
Paroxysmal AF, *n* (%)	11 (18.0)	47 (72.3)	<0.001	20 (43.5)	20 (43.5)	1
Current smoking, *n* (%)	27 (44.3)	18 (27.7)	0.052	18 (39.1)	18 (39.1)	1
Alcohol consumption, *n* (%)	17 (27.9)	7 (10.8)	0.015	12 (26.1)	4 (8.7)	0.12
SAHS, *n* (%)	1 (1.6)	0	0.3	2 (4.3)	0	0.312
COPD, *n* (%)	4 (6.6)	5 (7.7)	0.805	4 (8.7)	6 (13.0)	0.805
History of syncope, *n* (%)	1 (1.6)	1 (1.5)	0.964	2 (4.3)	0	0.312
Hypertension, *n* (%)	51 (83.6)	38 (58.5)	0.046	34 (73.9)	30 (65.2)	0.522
CAD, *n* (%)	11 (18.0)	9 (13.8)	0.52	10 (21.7)	12 (26.1)	0.73
History of MI, *n* (%)	2 (3.3)	1 (1.5)	0.522	2 (4.3)	0	0.312
History of PCI, *n* (%)	5 (8.2)	4 (6.2)	0.656	8 (17.4)	4 (8.7)	0.381
Myocarditis, *n* (%)	0	1 (1.5)	0.331	0	0	-
Diabetes, *n* (%)	9 (14.75)	11 (16.9)	0.52	8 (17.4)	10 (21.7)	0.71
History of stroke/TIA, *n* (%)	8 (13.1)	4 (6.2)	0.183	6 (13.0)	2 (4.3)	0.295
Dyslipidemia, *n* (%)	2 (3.3)	4 (6.2)	0.449	4 (8.7)	2 (4.3)	0.55
Symptomatic heart failure, *n* (%)	11 (18.0)	3 (4.6)	0.017	6 (13.0)	4 (8.7)	0.636
History of hyperthyreosis, *n* (%)	0	3 (4.6)	0.089	0	0	-
History of Hypothyroidism, *n* (%)	0	1 (1.5)	0.331	0	0	-
History of valve surgery, *n* (%)	0	1(1.5)	0.331	0	0	-

Data are presented as means ± standard deviations or medians (interquartile range), and counts (percentages).

ICE, Intracardiac echocardiography; AF, atrial fibrillation; BMI, body mass index; SAHS, sleep apnea hypopnea syndrome; COPD, chronic obstructive pulmonary disease; PAH, pulmonary hypertension; CHD, congenital heart disease; CAD, coronary artery disease; MI, myocardial infarction; PCI, percutaneous coronary intervention; TIA, transient ischemic attack.

**Table 2:** Laboratory test results of enrolled patients before and after propensity score matching.

Variables	Before matching			After matching		
	ICE (*n* = 61)	No-ICE (*n* = 65)	*P*	ICE (*n* = 46)	No-ICE (*n* = 46)	*P*
Heart rate (bpm)	80.0 (70.0, 98.0)	77.0 (64.0, 91.5)	0.312	74 (63, 86)	84 (70, 104)	0.053
Echocardiography						
IVSd (mm)	10 (9, 10)	9 (9, 10)	0.195	9 (8, 10)	9 (8, 10)	0.474
LVIDd (mm)	50 (47, 54)	50 (47, 53)	0.353	49.13 ± 3.77	50.26 ± 3.71	0.311
LVPWD (mm)	9 (9, 10)	9 (9, 10)	0.455	9 (8, 10)	9 (9, 9)	0.53
LVIDs (mm)	35 (33, 38)	33 (31.5, 36)	0.006	33 (32, 35)	33 (32, 38)	0.682
AO (mm)	31 (29, 33)	31 (29, 32)	0.399	31.26 ± 3.05	31.48 ± 2.81	0.803
LAD (mm)	44.16 ± 5.122	41.98 ± 5.201	0.019	43.39 ± 5.79	42.70 ± 5.44	0.677
FS (%)	30 (27.5, 32)	32 (30, 34)	<0.001	31.30 ± 2.82	31.52 ± 3.18	0.807
LVEF (%)	57 (53, 60)	60 (57, 63)	<0.001	58.78 ± 3.92	59.04 ± 4.87	0.842
RA > 40 mm, *n* (%)	14 (23.0)	8 (12.3)	0.116	5 (21.7)	5 (21.7)	1
WBC (×10^9^/L)	5.90 (4.95, 6.79)	6.21 (4.96, 7.81)	0.518	6.03 (5.44, 6.70)	6.28 (4.96, 7.42)	0.947
NEUT (%)	63.5 (57.1, 67.4)	64.0 (56.9, 71.8)	0.505	62.46 ± 7.06	65.17 ± 8.82	0.257
RBC (×10^9^/L)	4.47 (4.20, 4.90)	4.42 (4.06, 4.81)	0.257	4.54 ± 0.58	4.51 ± 0.59	0.838
Hb (g/L)	141.00 (127.50, 152,50)	134.00 (123.00, 147.00)	0.1	140.13 ± 17.86	138.61 ± 16.75	0.767
PLT (×10^9^/L)	196.00 (154.50, 223.50)	200.00 (159.50, 251.50)	0.21	200 (156, 220)	189 (161, 250)	0.86
CRP (mg/L)	0.65 (0.50, 3.21)	0.60 (0.50, 3.69)	0.899	0.65 (0.50, 3.64)	0.56 (0.50, 2.12)	0.698
TSH (pg/ml)	1.44 (1.08, 2.16)	1.64 (0.97, 2.65)	0.561	1.52 (1.07, 2.38)	1.57 (0.76, 2.36)	0.869
FT3 (pg/ml)	5.06 (4.54, 5.44)	4.78 (4.39, 5.20)	0.079	4.55 (4.16, 5.58)	5.04 (4.48, 5.41)	0.455
FT4 (pg/ml)	16.35 (15.52, 18.08)	16.76 (14.58, 18.47)	0.947	16.49 (15.74, 17.70)	17.37 (16.03, 18.78)	0.302
TNI (ng/ml)	0.012 (0.012, 0.012)	0.012 (0.012, 0.012)	0.221	0.012 (0.012, 0.012)	0.012 (0.012, 0.012)	0.590
BNP (pg/ml)	624.00 (384.50, 1,080.00)	325.00 (91.20, 552.00)	<0.001	483 (112, 902)	480 (91.2, 745)	0.717

Data are presented as means ± standard deviations or medians (interquartile range), and counts (percentages).

AO, aorta; BNP, brain natriuretic peptide; CRP, C-reactive protein; FS, fractional shortening; FT3, free triiodothyronine; FT4, free thyroxine; Hb, hemoglobin; IVSd, Interventricular septum thickness in diastole; LVIDd, left ventricular internal diameter in diastole; LVPWD, left ventricular posterior wall thickness in diastole; LVIDs, left ventricular internal diameter in systole; LAD, left atrial diameter; LVEF, left ventricular ejection fraction; NEUT, neutrophil count; PLT, blood platelet; RAD, right atrial diameter; RBC, red blood cell; TNI, Troponin I; TSH, thyroid-stimulating hormone; WBC, white blood cell.

### Procedural parameters of two groups

**Figure 3:**
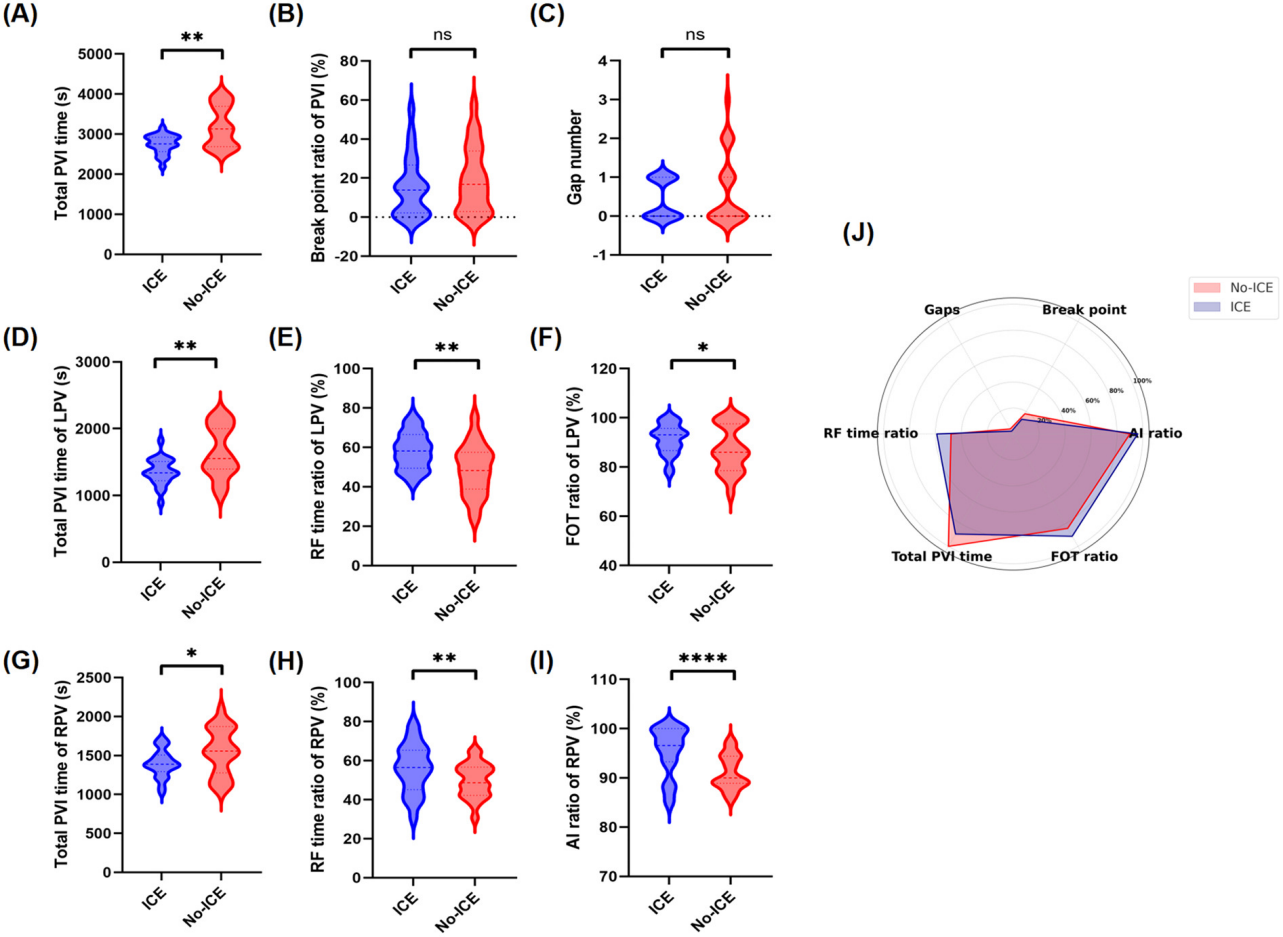
Comparison of six AIFV parameters between ICE and No-ICE groups. Violin plots show median values with 25th and 75th percentiles for **(A)** total PVI time (seconds), **(D)** PVI time of LPV (seconds), **(G)** PVI time of RPV (seconds), **(B)** break point ratio (%), **(C)** gap number, **(E)** RF time ratio for LPV (%), **(F)** FOT ratio for LPV (%), **(H)** RF time ratio for RPV (%), and **(I)** AI ratio for RPV (%). **(J)** Radar chart of six AIFV parameters. Statistical significance denoted by *p < 0.05, **p < 0.01, ***p < 0.001. AI, ablation index; FOT, force over time; LPV, left pulmonary vein; PVI, pulmonary vein isolation; RF, radiofrequency; RPV, right pulmonary vein.

Procedural characteristics are presented in Supplementary Table S1. PVI was achieved in all cases and performed better in the ICE group compared with the No-ICE group. Specifically, the ICE group exhibited shorter total PVI time compared to the No-ICE group [2,819 s (2,565 s, 2,953 s) vs. 3,153 s (2,696 s, 3,831 s); *p* = 0.006]. When isolating the left pulmonary vein, the ICE group required less time [1,347 s (1,221 s, 1,501 s) vs. 1,553 s (1,377 s, 2,098 s); *p* = 0.034], had a higher RF time ratio (58.81% ± 9.03% vs. 47.76% ± 13.56%; *p* = 0.002), and exhibited a higher effective FOT ratio (91.14% ± 5.90% vs. 84.53% ± 12.03%; *p* = 0.04). When isolating the right pulmonary vein, the ICE group required less time [1,388 s (1,237 s, 1,531 s) vs. 1,558 s (1,314 s, 1,872 s); *p* = 0.029], had a higher RF time ratio (59.29% ± 18.73% vs. 48.56% ± 9.71%; *p* = 0.021), and exhibited a higher effective AI ratio (96.10% ± 4.51% vs. 91.18% ± 3.88%; *p* < 0.001). Moreover, the ICE group had fewer gap occurrences and a lower break point ratio, but it did not reach statistical significance. The differences in these parameters between the two groups are presented in Figure 3. First-pass isolation, defined as achieving PVI with a single continuous ablation circle without additional touch-up ablations, was achieved in 37 of 46 patients (80.4%) in the ICE group compared to 30 of 46 patients (65.2%) in the No-ICE group (*p* = 0.092), with no significant difference. Multivariate regression analysis, adjusted for age, gender, AF type, and LAD, confirmed that ICE use was independently associated with improved procedural outcomes in the propensity score-matched cohort. Specifically, ICE use was associated with a significant reduction in total PVI time (*β* = −334.2 s, 95% CI: −567.8 to −100.6, *p* = 0.005), a higher RF time ratio (*β* = 10.8%, 95% CI: 6.2–15.4, *p* < 0.001), a higher effective AI ratio (*β* = 4.9%, 95% CI: 2.7–7.1, *p* < 0.001), and a higher effective FOT ratio (*β* = 6.6%, 95% CI: 2.1–11.1, *p* = 0.004), as shown in Supplementary Table S2.

### Follow-up outcomes of two groups

**Figure 4:**
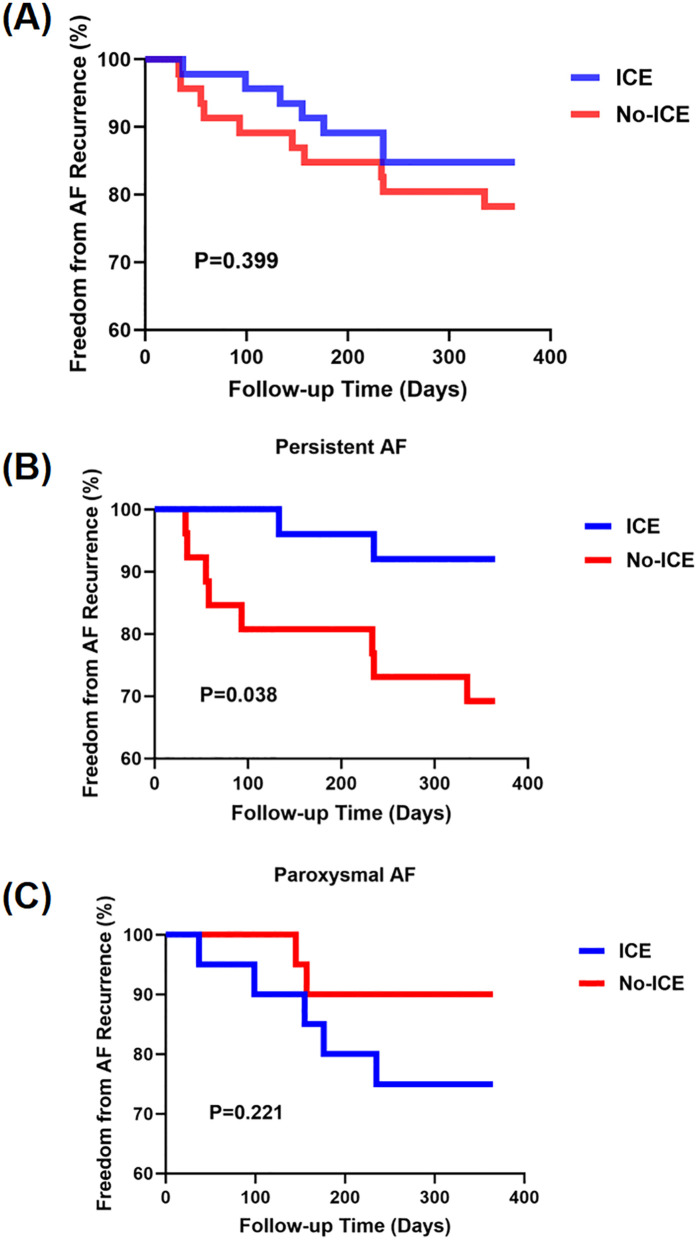
Kaplan–Meier survival curve for freedom from AF recurrence (in months) over 12 months post-PVI in ICE and No-ICE groups. **(A)** Overall survival analysis for both groups. **(B)** Subgroup analysis for persistent AF. **(C)** Subgroup analysis for paroxysmal AF.

After a median follow-up of 12 months, AADs were reinitiated in 7 patients (15.2%) in the ICE group and 10 patients (21.7%) in the No-ICE group following the 3-month blanking period due to AF recurrence, with no statistically significant difference between the groups (*p* = 0.399). To further investigate potential differences between AF subtypes, we performed a subgroup analysis. Among patients with persistent AF, the recurrence rate was significantly lower in the ICE group compared to the No-ICE group (7.7% vs. 30.8%, *p* = 0.038). However, no significant difference was observed in patients with paroxysmal AF (*p* = 0.221). The time-to-event analysis is illustrated in Figure 4.

## Discussion

This study provides the first detailed comparison of ICE-guided vs. No-ICE-guided PVI in AF patients, utilizing the novel AIFV system. Our findings demonstrate that ICE significantly enhances procedural efficiency, evidenced by reduced PVI time, and improves lesion quality, as shown by higher effective FOT and AI ratios. These results highlight ICE's technical advantages in optimizing ablation outcomes. The main finding of our study were shown in Graphical Abstract.

### ICE enhances procedural efficiency of PVI

During PVI, ICE provides real-time, high-resolution imaging of intracardiac structures, enabling precise localization of ablation sites (20). ICE assists physicians in visualizing the anatomical features of the left atrium, accurately identifying the pulmonary vein ostia, and ensuring optimal positioning of the ablation catheter to effectively target the intended areas. Additionally, ICE offers continuous real-time feedback during the ablation process, allowing clinicians to monitor the contact between the ablation electrode and cardiac tissue, assess lesion formation, and evaluate procedural effectiveness (21). This dynamic visualization capability empowers physicians to fine-tune electrode positioning and adjust ablation parameters as needed, enhancing procedural efficacy while minimizing the risk of damage to adjacent structures (22).

Our results indicate that ICE significantly decreases PVI and RF durations while enhancing the RF ratio. These results suggest that ICE not only improves procedural control but also minimizes time wasted on managing and closing gaps. Shortening ablation duration is particularly important for elderly AF patients, who often have multiple comorbidities, such as osteoporosis and sarcopenia, which limit their tolerance for prolonged procedures. Furthermore, extended procedure times can elevate the risk of thrombosis. The use of ICE contributes to a safer and more comfortable experience for AF patients.

### ICE improves quality of PVI

In research conducted by Medhat Farwati et al., 36% of patients experienced recurrence after their first ablation, whereas only 8% did so after a second procedure and ≥70% of patients reported significant improvement in their AF-related symptoms (23). The considerable disparity between the success rates of initial and repeated PVI highlights the urgent necessity to improve the quality of lesions formed during the first isolation attempt. It is widely recognized that factors such as lesion transmurality, durability, and continuity are essential measures of PVI quality, significantly influencing sustained control of atrial fibrillation in the long run (24). Among these factors, transmurality and durability rely on maintaining stable catheter-to-atrial wall contact with adequate force, as well as delivering consistent power for a sufficient duration.

A number of indices have been created through experimental models to evaluate how lesion depth and volume correlate with contact force, duration of ablation, and power settings. Nonetheless, in a clinical setting, achieving consistent catheter contact with the atrial wall can be quite difficult, influenced by elements like breathing, cardiac movement, and the manipulation of the catheter. The integration of ICE enhances the precision of atrial model construction, enabling finer control of catheter tip movement. This stability enables a steadier contact force, which may result in more effective lesions than intermittent contact (25). Our study demonstrated that the ICE group exhibited a higher effective FOT and AI ratios than the No-ICE group.

### Potential benefit of ICE beyond the efficacy and quality

ICE offers a favorable safety profile, which is particularly beneficial for elderly AF patients with comorbidities. Studies have shown that ICE-guided procedures, such as left atrial appendage closure, achieve high success rates (94.5%–97%) with lower complication rates (1.8% vs. 4.7% for TEE) and reduced contrast use (26). In our study, no complications were reported in either group, but the shorter ablation times with ICE (2,819 s vs. 3,153 s, *p* = 0.006) may further enhance safety by minimizing procedural risks, such as thrombosis, in vulnerable populations. These findings highlight ICE's potential to improve both safety and patient comfort during PVI.

### Mechanistic insights into ICE's benefits

The improvements in ablation parameters observed with ICE likely result from its dual contributions to enhanced visualization and catheter stability. ICE provides real-time, high-resolution imaging of intracardiac structures, such as the pulmonary vein ostia and left atrial anatomy, enabling precise localization of ablation targets. This improved visualization reduces unnecessary catheter movements, minimizing procedural time and enhancing efficiency, as evidenced by the shorter total PVI time (2,819 s vs. 3,153 s, *p* = 0.006) and higher RF time ratio (59.1% vs. 48.2%, *p* < 0.001) in the ICE group. Additionally, ICE offers continuous feedback on catheter-tissue contact, allowing operators to maintain stable contact force (≥5 g for >40% of the intervention, as reflected in the higher FOT ratio: 91.14% vs. 84.53%, *p* = 0.04). This stability is critical for achieving consistent, transmural lesions, as intermittent contact can lead to incomplete ablation. By combining precise anatomical guidance with real-time monitoring of catheter stability, ICE optimizes both the accuracy and effectiveness of PVI, contributing to the observed improvements in lesion quality metrics, such as the effective AI ratio (96.1% vs. 91.2%, *p* < 0.001).

### Cost implications of ICE use

While our study did not assess the cost of ICE-guided PVI, the adoption of ICE may involve higher upfront costs due to the need for specialized equipment and training. However, the observed reductions in PVI time (2,819 s vs. 3,153 s, *p* = 0.006) and improved lesion quality (e.g., higher AI ratio: 96.1% vs. 91.2%, *p* < 0.001) may lead to fewer redo procedures, potentially offsetting costs over time. Previous studies on ICE-guided left atrial appendage closure have reported reduced fluoroscopy and contrast use, which could further contribute to cost savings by minimizing complications and hospital resource utilization. Future studies should include cost-effectiveness analyses to better understand the economic implications of routine ICE use in AF ablation.

### Study limitations

This study has several limitations. First, while ICE significantly reduced the recurrence rate in the subgroup analysis of persistent AF, no significant reduction was observed in the overall cohort or the paroxysmal AF subgroup. These findings suggest that while ICE enhances technical aspects of PVI, its impact on long-term rhythm control remains inconclusive in this study, potentially due to limitations in sample size or follow-up duration. Second, Propensity score matching balanced key variables but could not account for unmeasured confounders like pulmonary vein anatomy. Third, Operators were unblinded to imaging modality, though AIFV analysis was blinded. Third, as a retrospective study, the sample size was determined by available data rather than a prospective calculation. While a *post hoc* power analysis confirmed sufficient power for the primary endpoint, prospective studies with predefined sample sizes could further validate these findings. Lastly, our study relied on industry-linked tools like AIFV system (accessible only via a Johnson & Johnson employee's account). While its algorithms are proprietary, the six metrics it evaluates are grounded in established ablation principles and have been previously described in peer-reviewed literature. However, we acknowledge that AIFV itself has not undergone independent external validation beyond industry-led studies. Had we taken measures to cross-validate with other methods, the results of this project could have been better validated. Larger, randomized trials with longer follow-up and validated tools are needed to confirm ICE's benefits for PVI. And future research should address the following questions: (1) Does ICE reduce long-term AF recurrence in larger, randomized trials with extended follow-up? (2) Are ICE's benefits more pronounced in specific patient subgroups, such as those with complex pulmonary vein anatomy or significant atrial fibrosis? (3) Is ICE cost-effective for routine use in AF ablation, considering both procedural costs and potential reductions in redo procedures? (4) How does ICE perform when combined with emerging ablation technologies, such as pulsed field ablation?

## Conclusion

ICE enhances the quality of lesions and the ablation efficiency of PVI in AF patients, as shown by the AIFV system.

## Data Availability

The raw data supporting the conclusions of this article will be made available by the authors, without undue reservation.

## References

[1] Hindricks G Potpara T Dagres N Arbelo E Bax JJ Blomström-Lundqvist C 2020 ESC guidelines for the diagnosis and management of atrial fibrillation developed in collaboration with the European Association for Cardio-Thoracic Surgery (EACTS). Eur Heart J. (2021) 42:373–498. 10.1093/eurheartj/ehaa612 32860505

[2] Calkins H Hindricks G Cappato R Kim Y-H Saad EB Aguinaga L 2017 HRS/EHRA/ECAS/APHRS/SOLAECE expert consensus statement on catheter and surgical ablation of atrial fibrillation. EP Europace. (2018) 20:e1–160. 10.1093/europace/eux274 PMC5834122 29016840

[3] Stewart MT Haines DE Miklavčič D Kos B Kirchhof N Barka N Safety and chronic lesion characterization of pulsed field ablation in a porcine model. Cardiovasc Electrophysiol. (2021) 32:958–69. 10.1111/jce.14980 PMC8048690 33650743

[4] Yu R Liu N Lu J Zhao X Hu Y Zhang J 3-dimensional transseptal puncture based on electrographic characteristics of fossa ovalis. JACC Cardiovasc Interv. (2020) 13:1223–32. 10.1016/j.jcin.2020.03.015 32438994

[5] Diaz JC Duque M Marin J Aristizabal J Niño C Bastidas O Intracardiac echocardiography-guided left atrial appendage occlusion. Arrhythm Electrophysiol Rev. (2024) 13:e03. 10.15420/aer.2023.29 38544808 PMC10964292

[6] Zhang G Cheng L Liang Z Zhang J Dong R Hang F Zero-fluoroscopy transseptal puncture guided by right atrial electroanatomical mapping combined with intracardiac echocardiography: a single-center experience. Clin Cardiol. (2020) 43:1009–16. 10.1002/clc.23401 32506504 PMC7462191

[7] Hu T Chen T Maduray K Han W Zhong J. Intracardiac echocardiography: an invaluable tool in electrophysiological interventions for atrial fibrillation and supraventricular tachycardia. Rev Cardiovasc Med. (2024) 25:191. 10.31083/j.rcm2506191 39076314 PMC11270097

[8] Liu X Lin R Peng X Wang X Li Y Liu X Visualization and mapping of the right phrenic nerve by intracardiac echocardiography during atrial fibrillation ablation. EP Europace. (2023) 25:1352–60. 10.1093/europace/euad012 PMC10105843 36857524

[9] Nielsen-Kudsk JE Berti S Caprioglio F Ronco F Arzamendi D Betts T Intracardiac echocardiography to guide watchman FLX implantation. JACC Cardiovasc Interv. (2023) 16:643–51. 10.1016/j.jcin.2022.10.024 36764917

[10] Ooms JF Hirsch A Von Der Thüsen JH Michels M Van Mieghem NM. Intracardiac echocardiography–guided biopsy in the work-up of an unexplained cardiac mass. JACC Cardiovasc Interv. (2021) 14:e297–9. 10.1016/j.jcin.2021.08.009 34656495

[11] Wang K Jin C Chen H Yang G Liu H Wang Z General anesthesia enhances lesion quality and ablation efficiency of circumferential pulmonary vein isolation. J Arrhythm. (2024) 40:76–82. 10.1002/joa3.12960 38333406 PMC10848594

[12] Cappato R Calkins H Chen S-A Davies W Iesaka Y Kalman J Updated worldwide survey on the methods, efficacy, and safety of catheter ablation for human atrial fibrillation. Circ Arrhythm Electrophysiol. (2010) 3:32–8. 10.1161/CIRCEP.109.859116 19995881

[13] Mesnier J Cepas-Guillén P Freixa X Flores-Umanzor E Hoang Trinh K O’Hara G Antithrombotic management after left atrial appendage closure: current evidence and future perspectives. Circ Cardiovasc Interv. (2023) 16:e012812. 10.1161/CIRCINTERVENTIONS.122.012812 37192309

[14] Katic J Borovac JA. Treatment of persistent left atrial appendage thrombus in patients with atrial fibrillation on adequate oral anticoagulation: pathways of care for all-comers and heart failure patients. Card Fail Rev. (2023) 9:e05. 10.15420/cfr.2022.28 37397240 PMC10311400

[15] Saw J Holmes DR Cavalcante JL Freeman JV Goldsweig AM Kavinsky CJ SCAI/HRS expert consensus statement on transcatheter left atrial appendage closure. Heart Rhythm. (2023) 20:e1–16. 10.1016/j.hrthm.2023.01.007 36990925

[16] Akhmerov A Ramzy D. Left atrial thrombus. N Engl J Med. (2022) 387:e8. 10.1056/NEJMicm2117229 35904480

[17] Della Rocca DG Magnocavallo M Gianni C Mohanty S Al-Ahmad A Bassiouny M Three-dimensional intracardiac echocardiography for left atrial appendage sizing and percutaneous occlusion guidance. Europace. (2023) 26:euae010. 10.1093/europace/euae010 38225176 PMC10823354

[18] Honarbakhsh S Schilling RJ Providencia R Dhillon G Bajomo O Keating E Ablation guided by STAR-mapping in addition to pulmonary vein isolation is superior to pulmonary vein isolation alone or in combination with CFAE/linear ablation for persistent AF. Cardiovasc Electrophysiol. (2021) 32:200–9. 10.1111/jce.14856 PMC8607469 33368766

[19] Verma A Haines DE Boersma LV Sood N Natale A Marchlinski FE Pulsed field ablation for the treatment of atrial fibrillation: PULSED AF pivotal trial. Circulation. (2023) 147:1422–32. 10.1161/CIRCULATIONAHA.123.063988 36877118 PMC10158608

[20] Ferro EG Alkhouli M Nair DG Kapadia SR Hsu JC Gibson DN Intracardiac vs transesophageal echocardiography for left atrial appendage occlusion with watchman FLX in the U.S. JACC Clin Electrophysiol. (2023) 9:2587–99. 10.1016/j.jacep.2023.08.004 37831030 PMC12337774

[21] Adams A Mahmood R Balaji N Dixit P Chandra S Weisman D. Real-world experience utilizing the nuvision 4D intracardiac echocardiography catheter for left atrial appendage closure. Sci Rep. (2024) 14:11937. 10.1038/s41598-024-60692-5 38789491 PMC11126603

[22] Tang GHL Zaid S Hahn RT Aggarwal V Alkhouli M Aman E Structural heart imaging using 3-dimensional intracardiac echocardiography: JACC cardiovascular imaging position statement. JACC Cardiovasc Imaging. (2025) 18:93–115. 10.1016/j.jcmg.2024.05.012 38970594

[23] Farwati M Amin M Saliba WI Nakagawa H Tarakji KG Diab M Impact of redo ablation for atrial fibrillation on patient-reported outcomes and quality of life. Cardiovasc Electrophysiol. (2023) 34:54–61. 10.1111/jce.15710 36259719

[24] Rotter M. Prospective validation of phased array intracardiac echocardiography for the assessment of atrial mechanical function during catheter ablation of atrial fibrillation. Heart. (2005) 92:407–9. 10.1136/hrt.2005.064295 PMC1860829 16501208

[25] Ranard LS Khalique OK Donald E Agarwal V Hamid N Hahn RT Transcatheter left atrial appendage closure using preprocedural computed tomography and intraprocedural 4-dimensional intracardiac echocardiography. Circ Cardiovasc Interv. (2021) 14:e010686. 10.1161/CIRCINTERVENTIONS.121.010686 34157847

[26] Korsholm K Jensen JM Nielsen-Kudsk JE. Intracardiac echocardiography from the left atrium for procedural guidance of transcatheter left atrial appendage occlusion. JACC Cardiovasc Interv. (2017) 10:2198–206. 10.1016/j.jcin.2017.06.057 28866042

