# Supplementary material for: Intracardiac echocardiography improves lesion quality and ablation efficiency of pulmonary vein isolation in atrial fibrillation patients: a propensity score-matched analysis
Source: Front Cardiovasc Med. 2025 Jul 1;12:1612181. doi: 10.3389/fcvm.2025.1612181 (PMC12259685; doi:10.3389/fcvm.2025.1612181)
Supplement: Supplementary file 1 [file Table1.doc]

**Supplemental Material**

**TABLE S1. Procedural parameters of the two groups After propensity score adjustment.**

|  | **ALL(n=92)** | **ICE(n=46)** | **No-ICE(n=46)** | **P** |
| --- | --- | --- | --- | --- |
| Total PVI time (s) | 2911.5 (2669.25, 3137.75) | 2819 (2565, 2952) | 3153 (2696, 3831) | 0.006 |
| Gap number | 0 (0, 1) | 0 (0, 1) | 0 (0, 1) | 0.094 |
| Break point ratio of PVI (%) | 14.6 (2.7, 27.4) | 13.9 (2.2, 26.7) | 16.8 (2.9, 33.9) | 0.288 |
| Total PVI time of LPV (s) | 1393 (1257.5, 1674.75) | 1347 (1221, 1501) | 1553 (1377, 2098) | 0.034 |
| RF time of LPV (s) | 757.5 (663, 892) | 804.96± 158.37 | 761.61± 157.90 | 0.358 |
| RF time ratio (%) | 53.86± 13.47 | 58.81± 9.03 | 47.76± 13.56 | 0.002 |
| RF points of LPV | 42 (37, 47) | 42.96± 8.16 | 40.39± 6.44 | 0.243 |
| FOT ratio of LPV (%) | 89.16± 8.48 | 91.14± 5.90 | 84.53± 12.03 | 0.04 |
| AI ratio of LPV (%) | 92.01± 5.66 | 93.32± 5.96 | 91.10± 5.73 | 0.124 |
| Gap＞8mm of LPVI, n (%) | 4 (4.34) | 2 (4.3) | 2 (4.3) | 1 |
| Drag point of LPVI (%) | 0 (0, 0) | 0 (0, 0) | 0 (0, 6.4) | 0.274 |
| Total PVI time of RPV (s) | 1478.5 (1293, 1681.75) | 1388 (1237, 1531) | 1558 (1314, 1872) | 0.029 |
| RF time of RPV (s) | 774.5 (676.5, 893.25) | 802.74± 177.76 | 765.17± 196.00 | 0.5 |
| RF time ratio (%) | 55.84± 12.74 | 59.29± 18.73 | 48.56± 9.71 | 0.021 |
| RF points of RPV | 44± 6.78 | 42± 7.80 | 40.96± 9.92 | 0.694 |
| FOT ratio of RPV (%) | 86.14± 11.14 | 88.05± 7.78 | 84.53± 12.03 | 0.245 |
| AI ratio of RPV (%) | 94.76± 5.05 | 96.10± 4.51 | 91.18± 3.88 | ＜0.001 |
| Gap＞8mm of RPVI, n (%) | 0 | 0 | 0 | - |
| Drag point of RPVI (%) | 0 (0, 0) | 0 (0, 0) | 0 (0, 0) | 0.975 |
| First-pass isolation, n (%) | 67 (72.82) | 37 (80.4) | 30 (65.2) | 0.092 |

**TABLE S2. Multivariate regression analysis of procedural outcomes in propensity score-matched cohort**

| **Outcome** | **Variable** | **β-value** | **95% CI** | **P** |
| --- | --- | --- | --- | --- |
| Total PVI time (s) | ICE Use (vs. No-ICE) | -334.2 | -567.8 to -100.6 | 0.005 |
|  | Age (years) | 5.2 | -3.1 to 13.5 | 0.219 |
|  | Gender (Male) | -45.3 | -278.9 to 188.3 | 0.702 |
|  | AF Type (Persistent) | 123.7 | -109.8 to 357.2 | 0.297 |
|  | LAD (mm) | 7.8 | -4.2 to 19.8 | 0.201 |
| RF time ratio (%) | ICE Use (vs. No-ICE) | 10.8 | 6.2 to 15.4 | ＜0.001 |
|  | Age (years) | -0.1 | -0.3 to 0.1 | 0.412 |
|  | Gender (Male) | 1.2 | -3.4 to 5.8 | 0.607 |
|  | AF Type (Persistent) | -1.9 | -6.5 to 2.7 | 0.419 |
|  | LAD (mm) | -0.2 | -0.5 to 0.1 | 0.189 |
| Effective AI ratio (%) | ICE Use (vs. No-ICE) | 4.9 | 2.7 to 7.1 | ＜0.001 |
|  | Age (years) | 0.0 | -0.1 to 0.1 | 0.872 |
|  | Gender (Male) | -0.5 | -2.7 to 1.7 | 0.652 |
|  | AF Type (Persistent) | -0.8 | -3.0 to 1.4 | 0.477 |
|  | LAD (mm) | -0.1 | -0.3 to 0.1 | 0.326 |
| Effective FOT ratio (%) | ICE Use (vs. No-ICE) | 6.6 | 2.1 to 11.1 | 0.004 |
|  | Age (years) | -0.1 | -0.3 to 0.1 | 0.384 |
|  | Gender (Male) | 0.9 | -3.7 to 5.5 | 0.698 |
|  | AF Type (Persistent) | -1.2 | -5.8 to 3.4 | 0.605 |
|  | LAD (mm) | -0.2 | -0.5 to 0.1 | 0.213 |

Note: Regression coefficients (β) represent the adjusted effect of each variable on the outcome. CI, confidence interval; ICE, intracardiac echocardiography; AF, atrial fibrillation; LAD, left atrial diameter; PVI, pulmonary vein isolation; RF, radiofrequency; AI, ablation index; FOT, force-over-time.
